# Chemical Composition of Mango (*Mangifera indica* L.) Fruit: Nutritional and Phytochemical Compounds

**DOI:** 10.3389/fpls.2019.01073

**Published:** 2019-10-17

**Authors:** Maria Elena Maldonado-Celis, Elhadi M. Yahia, Ramiro Bedoya, Patricia Landázuri, Nelsy Loango, Johanny Aguillón, Beatriz Restrepo, Juan Camilo Guerrero Ospina

**Affiliations:** ^1^Escuela de Nutrición y Dietética, Universidad de Antioquia, Medellín, Colombia; ^2^Facultad de Ciencias Naturales, Universidad Autónoma de Querétaro, Querétaro, Mexico; ^3^Facultad de Ciencias Agrarias, Universidad de Antioquia, Medellín, Colombia; ^4^Facultad de Ciencias de la Salud, Universidad del Quindío, Armenia, Colombia; ^5^Programa de Biología, Facultad de Ciencias Básicas y Tecnologías, Universidad del Quindío, Armenia, Colombia; ^6^Escuela Normal Superior del Quindío, Armenia, Colombia; ^7^Programa de Doctorado en Ciencias Biomédicas, Facultad Ciencias de la Salud, Universidad del Quindío, Armenia, Colombia

**Keywords:** *Mangifera indica*, mango, maturation, postharvest, nutrition, antioxidants, polyphenols, carotenoids

## Abstract

Mango fruit has a high nutritional value and health benefits due to important components. The present manuscript is a comprehensive update on the composition of mango fruit, including nutritional and phytochemical compounds, and the changes of these during development and postharvest. Mango components can be grouped into macronutrients (carbohydrates, proteins, amino acids, lipids, fatty, and organic acids), micronutrients (vitamins and minerals), and phytochemicals (phenolic, polyphenol, pigments, and volatile constituents). Mango fruit also contains structural carbohydrates such as pectins and cellulose. The major amino acids include lysine, leucine, cysteine, valine, arginine, phenylalanine, and methionine. The lipid composition increases during ripening, particularly the omega-3 and omega-6 fatty acids. The most important pigments of mango fruit include chlorophylls (*a* and *b*) and carotenoids. The most important organic acids include malic and citric acids, and they confer the fruit acidity. The volatile constituents are a heterogeneous group with different chemical functions that contribute to the aromatic profile of the fruit. During development and maturity stages occur important biochemical, physiological, and structural changes affecting mainly the nutritional and phytochemical composition, producing softening, and modifying aroma, flavor, and antioxidant capacity. In addition, postharvest handling practices influence total content of carotenoids, phenolic compounds, vitamin C, antioxidant capacity, and organoleptic properties.

## Introduction

**Figure 1:**
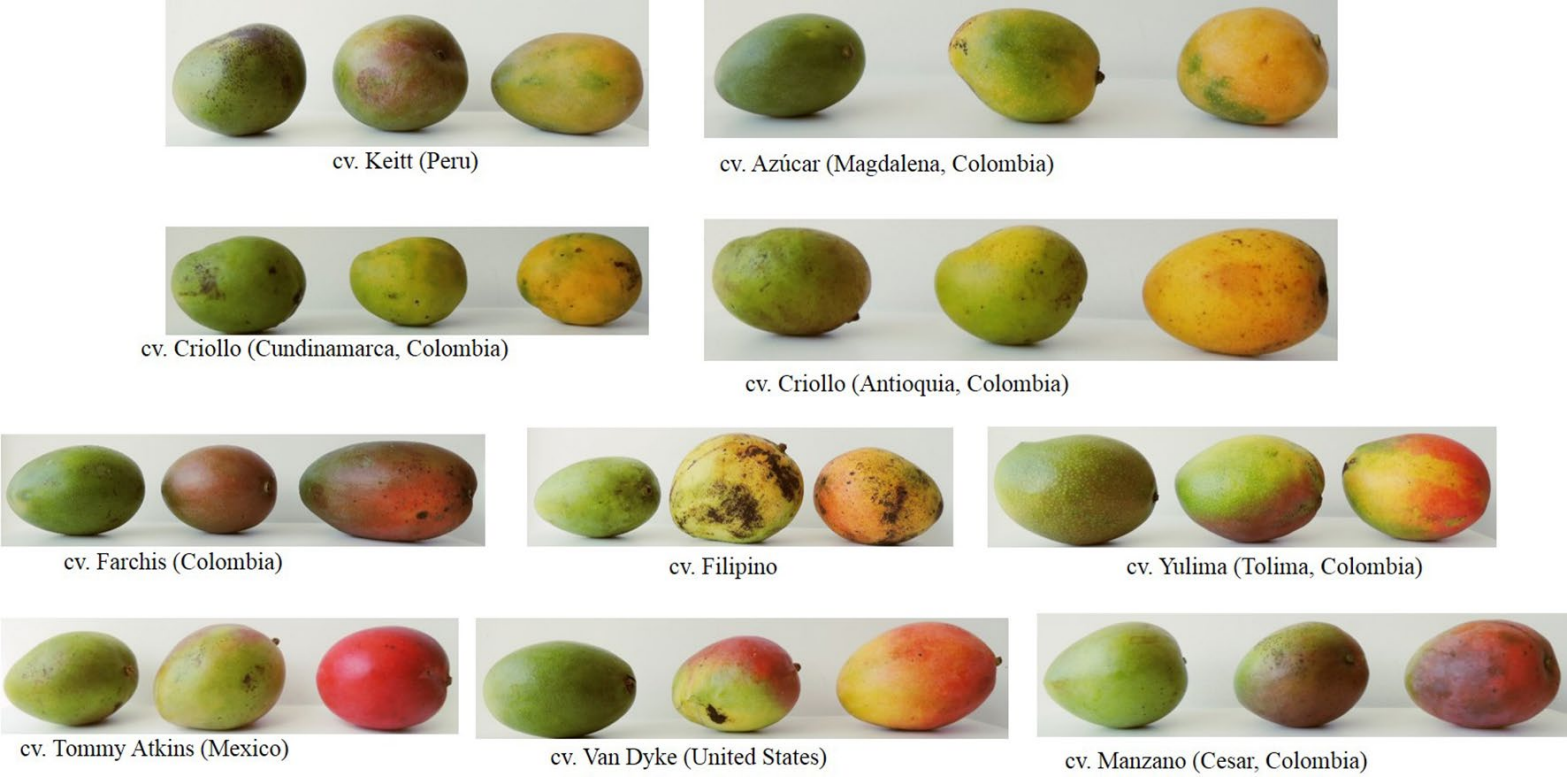
Photographs of mango cultivars in different ripening stages.

This manuscript presents a compilation of updated information on the nutritional composition of different mango varieties (**Figure 1**), as well as their main phytochemical components, useful for human nutrition, health, and other applications for agricultural, pharmaceutical, and food industries and the changes of these components during development and postharvest. This knowledge should contribute to control fruit deterioration, greater use, and valorization of the fruit.

The review consists of four parts for better understanding of the reader. The first part corresponds to the description on the nutritional content of mango fruit based on its macronutrients (carbohydrates, lipids and fatty acids, proteins and amino acids, and organic acids) and micronutrients (vitamins and minerals). The second part continues with the analysis of the most relevant phytochemical compounds identified in mango fruit (phenolic acids, flavonoids, and pigments such as chlorophyll and carotenoids) not only in the edible portion of the fruit but also in the seed and skin, in order to show that by-products of the mango fruit can be used with potential benefit for health and industry.

In the third part, the changes on nutritional and phytochemical composition of mango fruit during the development and ripening are explained. It is well known that chemical components of mango vary according to the region of planting, cultivar, cultural practices, and nutritional conditions of the plant. However, the mango fruit development and ripening involve a series of biochemical, physiological, and structural controlled changes that affect the content of nutrients, phytochemical compounds, and the organoleptic characteristics leading to a soft, ripe, and edible mango fruit with desirable attributes for consumer acceptance.

Finally, taking into account that mango is a climacteric fruit that continues the ripening processes after detachment from the parent plant, attributed to the increases in the rate of respiration and ethylene production (Tharanathan et al., 2006), several postharvest handling processes can reduce their postharvest life and also the fruit composition, leading to losses in terms of quality and quantity, which can be considerably reduced by applying adequate and improved strategies and technologies to prolong the shelf life of mango fruit.

## Nutritional Composition

Mango fruit is an important source of macronutrients such as carbohydrates, lipid and fatty acids, protein and amino acids, and organic acids. Also, mango has micronutrients such as vitamins and minerals and, finally, non-nutrients compound such as phenolic compounds, flavonoids and other polyphenols, chlorophyll, carotenoids, and volatile compounds. The energy value for 100 g of the pulp ranges from 60 to 190 kcal (250–795 kJ), being an important fruit for the human diet (**Table 1**). The nutritional, non-nutritional, and water contents of mango fruit vary depending of the cultivar and several preharvest and postharvest factors. For example, according to the United States Department of Agriculture (USDA) data of nutrient report, the mature mango pulp of Haden, Kent, Keitt, and/or Tommy Atkins varieties present 83.4 g of water per 100 g of fresh fruit, while the cultivar Azúcar from Colombia contain 79.3 g (Corrales-Bernal et al., 2014).

**Table 1:** Proximal composition analysis of mature mango fruit (*Mangifera indic a* L.) (taken in part from Tharanathan et al., 2006).

Parameter	Content (g per 100 g of fruit dry weight basis)
Water	78.9–82.8
Ashes	0.34–0.52
Total lipid	0.30–0.53
Total protein	0.36–0.40
Total carbohydrate	16.20–17.18
Total dietary fiber	0.85–1.06
Energy (kcal)	62.1–190

### Macronutrients

#### Carbohydrates

Ripened mango fruit is a major source of sugars (glucose, fructose, and sucrose) and other carbohydrates such as starch and pectins (Bello-Pérez et al., 2007). All these are significant compounds from a nutritional and flavor aspect. The fruit flesh of ripe mango contains about 15% of total sugars. Fructose is the major monosaccharide during the preclimateric phase (Bernardes et al., 2008), while sucrose is the principal sugar in ripe mango fruit (Instituto Colombiano de Bienestar Familiar (ICBF), 2015; Saleem-Dar et al., 2016; United States Department of Agriculture, Agricultural Research Service, 2018).

The USDA Nutrient Database (United States Department of Agriculture, Agricultural Research Service, 2018) reports that the total carbohydrate and sugar contents of Tommy Atkins, Haden, Kent, and Keitt cultivars per 100 g fruit is 14.98 and 13.66 g, respectively (sucrose, 6.97 g; glucose, 2.01; and fructose, 4.68 g), and 1.6 g of dietary fiber/100 g of fruit. The table of food composition from Colombia (Instituto Colombiano de Bienestar Familiar (ICBF), 2015) presented the content of glucose, fructose, sucrose, starch, and pectin in mango pulp (3.9, 1.0, 8.8, 1.8, and 8.2 g/100 g of pulp, respectively) and 2.6 g of dietary fiber. In African mango cultivars, the total sugar content varied between 10.5% and 32.4% (Othman and Mbogo, 2009), while the interval diminishes to 10–12% in Sudanese varieties (Nour et al., 2011). In general, many cultivars of mango contain sucrose, fructose, and glucose in order of highest to lowest (Bello-Pérez et al., 2007).

Starch is the most important from a quantitative point of view in the unripe mango fruit. During ripening, starch is hydrolyzed to glucose (Derese et al., 2017). After phosphorylation, glucose phosphate enters the hexose phosphate pool and fuels a “futile cycle” of sucrose synthesis and degradation (Geigenberger and Stitt, 1991) that controls the content of glucose, fructose, and sucrose, as it was found in kiwifruit (Moscatello et al., 2011). Thus, during ripening, glucose, fructose, and sucrose generally increase (Bernardes et al., 2008). The increase of these monosaccharides and disaccharides during maturation has been observed in cultivars Baladi (Sharaf et al., 1989), Haden (Castrillo et al., 1992), Alphonso (Yashoda et al., 2005), Dashehari (Kalra and Tandon, 1983), Keitt (Medlicott et al., 1986), and Tommy Atkins (Tasneem, 2004).

Pectin is a structural carbohydrate abundant in mango pulp and is considered an important component as a gelling sugar. When fruit is unripe, pectin is accumulated, but during ripening, its molecular weight decreases (Bello-Pérez et al., 2007; Saleem-Dar et al., 2016); this is attributed to the activity of hydrolysis of pectin enzymes in this stage (Prasanna et al., 2004).

#### Proteins and Amino Acids

Mango, like many fruits, has a low protein content with respect to the other macronutrients; by example, mango pulp from Colombia contributes to 0–0.6% of protein (Instituto Colombiano de Bienestar Familiar (ICBF), 2015; Corrales-Bernal et al., 2014), while in Peru, mango contains 1.5 to 5.5% total protein; in other cultivars like Java, mango has 1–2%, and in India, cultivars present low contents of total protein (0.5–1%) (Saleem-Dar et al., 2016).

The amino acid composition also varies among cultivars and maturation levels (Augustin et al., 1978). The amino acids alanine, arginine, glycine, serine, leucine, and isoleucine have been detected in considerable amounts in the ripe state, while all other amino acids are present in trace amounts (Tharanathan et al., 2006). **Table 2** presents the content of amino acids described by the United States Department of Agriculture, Agricultural Research Service, (2018).

**Table 2:** Amino acid composition in edible portion of mango fruit (United States Department of Agriculture, Agricultural Research Service, 2018).

Amino acid	Content of g/100 g
Isoleucine	0–0.029
Leucine	0–0.050
Lysine	0–0.066
Methionine	0–0.008
Phenylalanine	0–0.027
Tyrosine	0–0.016
Tryptophan	0–0.013
Threonine	0–0.031
Valine	0–0.042
Histidine	0–0.019
Arginine	0–0.031
Alanine	0–0.082
Aspartic acid	0–0.068
Glutamic acid	0–0.096
Glycine	0–0.034
Proline	0–0.029
Serine	0–0.035

#### Lipids and Fatty Acids

Lipids are nutrients present in small quantities in mango pulp; however, the seed and the peel have been considered a source of fatty acids and are compared with cocoa butter (**Table 3**). These fatty acids are useful mango by-products and can be used in the pharmaceutical and food industries.

**Table 3:** Content of fatty acids in mango fruit.

Carbon skeleton	Common name	Variety	Content	Part of the fruit
16:0^a^	Palmitic acid	Malaysia Mixed Egypt Manila Mexico Kaew Thailand 4 varieties Kenya	6.95–10.93 5.8 9.29 5.4 4.87–10.57	Seed
18:0^a^	Stearic acid	Malaysia Mixed Egypt Manila Mexico Kaew Thailand 4 varieties Kenya	32.8–47.62 38.3 39.07 46.6 24.22–32.80	Seed
20:0^a^	Arachidic acid	Malaysia Mixed Egypt Manila Mexico Kaew Thailand 4 varieties Kenya	1.77–2.43 — 2.48 1.7 0.67–1.64	Seed
24:0^a^	Lignoceric acid	—	—	Seed
18:1 (∆^9^)^a^	Oleic acid	Malaysia Mixed Egypt Manila Mexico Kaew Thailand 4 varieties Kenya	37.01-47.28 46.1 40.81 41.1 46.37–58.59	Seed
18:2 (∆^9,12^)^a^	Linoleic acid	Malaysia Mixed Egypt Manila Mexico Kaew Thailand 4 varieties Kenya	3.66–6.87 8.2 6.06 3.8 6.73–10.4	Seed
18:3 (∆^9,12,15^)^a^	α-Linoleic acid	—	—	Seed
14:0^b^	Myristic acid	Alphonso Pairi Kent	174.29, 231.21 74.03, 295.16 40.57, 323.9	Pulp, peel
16:0^b^	Palmitic acid	Alphonso Pairi Kent	1,933.43, 2,682.16 896, 3,460.13 560.88, 2,883.29	Pulp, peel
18:0^b^	Stearic acid	Alphonso Pairi Kent	75.63, 123.57 33.36, 238.57 29.76,116.39	Pulp, peel
20:0^b^	Arachidic acid	Alphonso Pairi Kent	19.01, 29.21 7.2, 55.24 3.2, 32.56	Pulp, peel
22:0^b^	Behenic acid	Alphonso Pairi Kent	24.90, 43.83 8.88, 55.38 3.67, 43.73	Pulp, peel
24:0^b^	Lignoceric acid	Alphonso Pairi Kent	35.85, 86.16 27.04, 1,17.24 24.88, 71.15	Pulp, peel
16:1, *n*−7^b^	Palmitoleic acid	Alphonso Pairi Kent	2,881.90, 1,986.59 599.84, 533.59 314.28, 1,527.72	Pulp, peel
16:1, *n*−5^b^	11-Hexadecenoic acid	Alphonso Pairi Kent	146.22, 119.07 51.49, 58.01 22.42, 147.05	Pulp, peel
17:1, *n*−7^b^	10-Heptadecenoic acid	Alphonso Pairi Kent	11.82, n.d. 8.76, n.d. 3.76, n.d.	Pulp, peel
18:1, *n*−9^b^	Oleic acid	Alphonso Pairi Kent	856.59, 2,376.3 761.79, 2,847.25 261.3, 778.48	Pulp, peel
18:1, *n*−7^b^	11-Octadecenoic acid	Alphonso Pairi Kent	646.48, 480.59 248.78, 321.16 176.61, 282.14	Pulp, peel
20:1, *n*−9^b^	11-Eicosenoic acid	Alphonso Pairi Kent	6.57, 10.01 2.39, 10.49 n.d., n.d.	Pulp, peel
16:2, *n*−4	9,12-Hexadecadienoic acid	Alphonso Pairi Kent	33.86, n.d. 17.71, n.d. 16.09, n.d.	Pulp, peel
18:2, *n*−6	Linoleic acid	Alphonso Pairi Kent	83.58, 422.83 139.44, 1,956.03 80.05, 1,277.41	Pulp, peel
18:2, *n*−3	9,15-Octadecadienoic acid	Alphonso Pairi Kent	61.58, n.d 20.24, n.d. 20.93, n.d.	Pulp, peel
7:2, *n*−3	Hepta-2,4(*E*,*E*)-dienoic acid	Alphonso Pairi Kent	698.01, 265.93 662.32, 1,152.72 835.33, 352.98	Pulp, peel
18:3, *n*−3	Linolenic acid	Alphonso Pairi Kent	840.37, 1,149.88 522.23, 1,991.68 408.42, 1,201.18	Pulp, peel

n.d., not detected.

a (Jahurul et al., 2015).

b µg/g tissue (Desphande et al., 2016).

The prominent fatty acids found in the mango kernel are palmitic, stearic, oleic, and linoleic. Lignoceric, arachidic, linolenic, and behenic acids are present in lower concentrations (**Table 3**) (Jahurul et al., 2015). The content of triglycerides determined in blend of mango seed was 11% to 38.8% of 1,3-dipalmitoyl-2-oleoyl-glycerol (POP), 22.1% to 36.9% of 1,3-distearoyl-2-oleoyl-glycerol (SOS), and 15.4% to 16.2% of 1-palmitoyl-3-stearoyl-2 oleoyl-glycerol (POS) (Jahurul et al., 2014a). It has been reported that the incorporation of SOS-rich fats in chocolate products increases the solid fat content, leading to the inhibition of fat bloom and reducing the tempering time (Reddy and Prabhakar, 1994; Maheshwari and Reddy, 2005). Therefore, SOS-rich fractions could be used as an ingredient for the production of temperature-resistant hard butter, which is particularly useful in countries with high temperatures (Jahurul et al., 2014c); thus, mango seed fat is a potential cocoa butter alternative (Solís-Fuentes and Durán-de-Bazúa, 2004; Sonwai et al., 2012; Jahurul et al., 2014b; Jahurul et al., 2014c).

The fatty acid content in the peel and pulp has been also studied in different varieties of mango such as Malgoa, Totapuri, Benishan, Sundari, and Neelam and was found to range from 0.75% to 1.7% in the skin and 0.8% to 1.36 in the flesh (Pathak and Sarada, 1974), with the triglycerides being the major components of the pulp, while monoglycerides and diglycerides are minor components (Selvaraj et al., 1989). In the cultivars Alphonso, Pairi, and Kent, 17 different fatty acids were identified and quantified during development and ripening of mango fruit with an increase of unsaturated fatty acids and an omega-6/omega-3 ≤ 1 at the ripe stage, which suggests that mango fruit is a good source of essential fatty acids (**Table 3**) (Desphande et al., 2016). This increase in fatty acid content during maturation has also been observed in Harumanis, Kalabau, Stam Panjang, African Bush, Fazil, and Kanchamithia varieties (Saleem-Dar et al., 2016).

Bandyopadhyay and Gholap (1973) showed the association between the intensity of aroma and flavor and determined the ratio of palmitic–palmitoleic acid in ripening mango pulp to apply as an index of aroma and flavor of mangoes. When this ratio is greater or less than 1, the fruit has a mild or strong aroma and flavor, respectively. It has been proposed that fatty acids are probably precursors for the biosynthesis of lactones. These compounds are important in the research on flavor biochemistry of foods (Desphande et al., 2016).

#### Organic Acids

Organic acids are characterized by weak acidic properties. These compounds may have low molecular weight such as oxalic and citric acids or too high molecular weight like humic acids with aromatic nuclei composed of carboxylic and phenolic functional substituents (Richter et al., 2007). Organic acids are necessary for aerobic metabolism and as flavor constituents that contribute to fruit quality, organoleptic properties, and fruit acidity (Vallarino and Osorio, 2019).

Organic acid compounds have been identified in different varieties of mango, and the content depends on acid synthesis, degradation, utilization, compartmentation, and external factors such as temperature, light, fertilization, water supply, and other plant management practices (Vallarino and Osorio, 2019).

Fruit acidity of mango is attributed mainly to the content citric and malic acids (Matheyambath et al., 2016), although other common organic acids from the tricarboxylic acid cycle have been reported in mango fruit including citric, oxalic, succinic, malic, and pyruvic as well as tartaric, muconic, galipic, glucuronic, and galacturonic acids; of these, citric is the major organic acid [0.13% to 0.71% fresh weight (FW)] (Shashirekha and Patwardhan, 1976; Sarker and Muhsi, 1981; Medlicott and Thompson, 1985; Tharanathan et al., 2006). For example, citric and malic acids are the major organic acids in Keitt mango, whereas α-ketoglutaric, ascorbic, oxalic, and tartaric acids are found in lower concentrations (Medlicott and Thompson, 1985; Medlicott et al., 1986). In mangoes from Badami, citric acid presents the highest concentration, but succinic and malic acids were also detected (Shashirekha and Patwardhan, 1976). A similar profile was described in Fazli variety (pyruvic, citric, succinic, oxalic, and malic acids); in addition to those, tartaric acid was determined in Zardalu mangoes (Kumar et al., 1993). On the contrary, mango cultivars from Africa present a moderate content of citric acid (0.2% to 1.3%) (Othman and Mbogo, 2009).

### Micronutrients

#### Vitamins

The nutrient database of USDA (National Nutrient Database for Standard Reference) reported the values of water-soluble and fat-soluble vitamins analyzed from Tommy Atkins, Keitt, Kent, and Haden cultivars (**Table 4**). Vitamin C and vitamin A are dominant, suggesting that regular consumption of mango fruit can provide the necessary dietary requirements of these vitamins (WHO/FAO, 2003).

**Table 4:** Vitamin composition in 100 g of edible portion of mango fruit (United States Department of Agriculture, Agricultural Research Service, 2018).

Vitamin	Value per 100 g
Ascorbic acid (Vit C)	13.2–92.8 mg
Thiamine (Vit B1)	0.01–0.04 mg
Riboflavin (Vit B2)	0.02–0.07 mg
Niacin (Vit B3)	0.2–1.31 mg
Pantothenic acid (Vit B5)	0.16–0.24 mg
Pyridoxine (Vit B6)	0.05–0.16 mg
Folate total	20–69 µg
Folic acid	0 µg
Folate food	20–69 µg
B12	0.00 mg
Vitamin A	54 µg
Vitamin E (α-tocopherol)	0.79–1.02 mg
Vitamin K	4.2 µg

Great variations exist in vitamin C content, fluctuating from 9.79 to 186 mg/100 g of mango pulp (Vazquez-Salinas and Lakshminarayana, 1985; Manthey and Perkins-Veazie, 2009; Wongmetha and Ke, 2012; ICBF, 2015; Matheyambath et al., 2016; USDA, 2018). The cultivars Kent, Tommy Atkins, and Keitt from Mexico, Peru, Brazil, and Ecuador; Haden from Peru and Mexico; and Ataulfo from Mexico were analyzed by Manthey and Perkins-Veazie (2009) who reported that the average vitamin C level over all harvest locations were 19.3, 24.7, 25.6, 31.0, and 125.4 mg/100 g of pulp in the Tommy Atkins, Keitt, Kent, Haden, and Ataulfo cultivars, respectively (Manthey and Perkins-Veazie, 2009). The higher vitamin C concentration in Ataulfo was comparable with the concentrations in Ubá cultivar from Brazil (77.7 mg/100 g FW) (Ribeiro et al., 2007). Sellamuthu et al., (2013) analyzed six African varieties (Tommy Atkins, Zill, Peach, Sabre, Rosa, and Phiva) and found that vitamin C content oscillated from 50.71 to 17.01 mg/100 g FW, being significantly higher (*P* < 0.05) in cv. Sabre and lower (*P* < 0.05) in cv. Tommy Atkins; and for the Palmer cultivar from Brazil, it was 40.9 mg/100 g FW (Valente et al., 2011). These variations are attributed to several different preharvest and postharvest factors, which all can influence the synthesis.

Vitamin C content changes during ripening; its content is higher in less ripe mango fruit compared with fully ripe mango (Matheyambath et al., 2016). Vitamin C decreases quickly 5 to 7 weeks after fruit setting and when ripe fruit is stored at room temperature (Yahia, 2011; Ibarraz-Garza et al., 2015). The vitamin C decrease may be due to the involvement of different metabolic pathways such as ethylene, oxalate, and tartrate biosynthesis because vitamin C is a coenzyme of their respective enzymes (Singh et al., 2011). In the analysis of vitamin C concentration in the pulp of Keitt, Sensation, and Xiangya mango cultivars from China at different stages (green and ripe mangoes), it was observed that the fruit pulp showed an important decrease during ripening (Keitt, 163.94 to 46.87 mg ascorbic acid equivalent (AAE)/100 g; Sensation, 176.03 to 29.34 mg AAE/100 g; and Xiangya, 160.35 to 30.84 mg AAE/100 g) (Hu et al., 2018).

The content of vitamin A in the fruit varies from 1,000 to 6,000 IU (Matheyambath et al., 2016). Thus, mango consumption is very important, especially for those regions where there is a deficiency of vitamin A (Muoki et al., 2009). Consumption of a single fruit (around 300 g) would supply 15–69 retinol equivalents (REs)/day depending on the cultivar. This would correspond to 11.5% of REs requirement per day for both teenagers and adults. For example, the consumption of three times per day of Tommy Atkins would provide more than 50% of the daily requirements of women and children aged 3–6 years (Muoki et al., 2009).

Similar conclusions were reported by Nana et al. (2005, 2006) who indicated that intervention strategies with mango improve the vitamin A intake by 50% and serum retinol concentrations by 26% of children (2 to 3 years) in Western Africa, over a 15-week period where mangoes are consumed little or nothing due to low seasonal availability.

The E and K vitamins are found in minor quantities (**Table 4**), while vitamin D has not been detected in any cultivars until now (ICBF, 2015; Saleem-Dar et al., 2016; USDA, 2018). The content of vitamin E is commonly low or moderate in fruit and can occur in tocopherols α-, β-, λ-, and γ-T; its corresponding tocotrienols are α-, β-, λ-, and γ-T3. The most biologically active form is α-tocopherol. In Ataulfo cultivar from Mexico, it was determined that fresh-cut mangoes contain 1.33 mg/100 g FW (Robles-Sanchez et al., 2009), which is greater than that informed by the USDA Nutrient Database (USDA, 2018) for Tommy Atkins, Kent, Keitt, and Haden.

Vitamin E increases from green mature stage to mature stage in Tommy-Kent mangoes, while in Tommy Atkins and Dasherai cultivars, the content of vitamin E is high at the unripe stage but later decreased (Barbosa Gámez et al., 2017; Singh et al., 2011). These changes in vitamin E content may in part be explained by the fact that vitamin C contributes to the biosynthesis of the oxidized form of vitamin E, tocopheroxyl radical, leading to the production of α-tocopherol (Méne-Saffrané, 2018). The opposite also occurs; that is, when vitamin C decreases, α-tocopherol content also decreases (Joas et al., 2009).

The vitamin B complex of mango fruit refers to water-soluble enzyme cofactors and their derivatives, which participate in different metabolic processes in plants and in humans. The vitamins included are thiamin (B1); riboflavin (B2); niacin (B3); pantothenic acid (B5); pyridoxine, pyridoxal, and pyridoxamine (B6); biotin (B8); and folate or folic acid (B9), except for biotin all the others vitamins, have been found in mango fruit (Saleem-Dar et al., 2016). All these vitamins B are important for proper human nutrition because humans are not able to synthesize these micronutrients. These vitamins can be also affected by several preharvest and postharvest factors, as well as the maturity stage because vitamin B synthesis is associated with the state of differentiation of cells (Aslam et al., 2010).

#### Minerals

According to the Recommended Daily Allowance (RDA) recommended levels by the National Research Council of USA (1989), mango may provide enough amount of essential minerals for human health, such as calcium, iron, magnesium, phosphorus, potassium, sodium, zinc, copper, manganese, and selenium. **Table 5** shows the essential mineral contents of Tommy Atkins, Keitt, Kent, and/or Haden (USDA, 2018) and Colombian cultivars (ICBF 2015). The major essential minerals that mango pulp contributes are K, P and Ca, while the levels of Na, Zn, and Fe were the lowest, and the seeds and peels contain significantly higher levels than does the pulp in the following order: Ca > K > Mg > Na > Fe > Mn > Zn > Cu (Njiru et al., 2014).

**Table 5:** Mineral composition in edible portion of mango fruit.

Mineral	Value (mg) per 100 g^a^	Value (mg) per 100 g^b^
Calcium	7–16	9–21
Iron	0.09–0.41	0.1–0.9
Magnesium	8–19	10–38
Phosphorus	10–18	19–23
Potassium	120–211	147–617
Sodium	0–3	0–4
Zinc	0.06–0.15	0–0.1
Copper	0.04–0.32	n.d.
Manganese	0.03–0.12	1.6–18.2
Selenium	0–0.6	n.d.

n.d., not determined.

a United States Department of Agriculture, Agricultural Research Service, (2018).

b Instituto Colombiano de Bienestar Familiar (ICBF), (2015).

## Phytochemical Components

### Phenolic Acids

Phenolic acids are plant secondary metabolites that form part of human diet and are of significant importance because of their biological abilities and health benefits (Brglez et al., 2016; Yahia et al., 2017). Mango pulp includes the two major categories of phenolic acids in plants, hydroxybenzoic and hydroxycinnamic acid derivatives. These phenolic acids may be present free or conjugated forms with glucose or quinic acid (Mattila and Kumpulainen, 2002, Burton-Freeman et al., 2017). The hydroxybenzoic acids that have been detected in the mango pulp are gallic, vanillic, syringic, protocatechuic, and *p*-hydroxybenzoic acids, while the hydroxycinnamic acid derivatives are *p*-coumaric, chlorogenic, ferulic, and caffeic acids (Masibo and Qian, 2008; Ediriweera et al., 2017). The content and characteristics of phenolic acids depend on the cultivar, crop, and ripening stage (Corrales-Bernal et al., 2014, Burton-Freeman et al., 2017).

Different phenolic acids were identified in the flesh and skin of nine mango varieties cultivated in China (Abbasi et al., 2015). The highest phenolic acid in 100 g FW of pulp was ferulic acid (33.75 mg), followed by protocatechuic (0.77 mg), chlorogenic (0.96–6.20 mg), gallic (0.93–2.98 mg), vanillic (0.57–1.63 mg), and caffeic acids (0.25–0.10 mg) (Abbasi et al., 2015). Similarly, the major phenolic acids in Ataulfo mango from Mexico were protocatechuic acid (0.48–1.1 mg/100 g dry weight (DW)), vanillic acid (16.9–24.4 mg/100 g DW), gallic acid (94.6–98.7 mg/100 g DW), and chlorogenic acid (28–301 mg/100 g DW) (Palafox-Carlos et al., 2012a; Palafox-Carlos et al., 2012b). Contrary to these studies, Corrales-Bernal et al. (2016) and Kim et al. (2009) found that the major phenolic acid in mango pulp of Azúcar and Tommy Atkins varieties was gallic acid; however, these authors were unable to quantify the other well-known phenolic acids (*p*-hydroxybenzoic, *p*-coumaric, and ferulic acids) because of low concentration. The peel extracts of the mango cultivars Ataulfo, Keitt, Osteen, and Sensation have been found to have high concentrations of phenolic acids and derivatives such as gallic, syringic, methyl digallate ester, methyl gallate, gallotannins, galloyl glucose, theogallin, protocatechuic, and ferulic acid (Gómez-Caravaca et al., 2015; López-Cobo et al., 2017; Pacheco-Ordaz et al., 2018).

Hu et al. (2018) recently identified tentatively 34 compounds as derivatives of phenolic acids including gallotannins and quercetin derivatives, reporting for first time the detection of rosmarinic acid in mango fruit in different stages of ripeness, both in the peel and in the pulp. All the determinations were done by ultra-performance liquid chromatography in combination with electrospray ionization and quadrupole time-of-flight mass spectrometry (UPLC–ESI–QTOFMS).

Several authors, using different methods such as high-performance liquid chromatography (HPLC)/ESI–MS (Berardini et al., 2003) and HPLC–photodiode array (PDA)–MS (Ramirez et al., 2013) to detect and identify other derivatives of phenolic compounds. Berardini et al. (2003) have detected in the peel of mango Tommy Atkins 18 gallotannins (1.4 mg/g dry matter (DM) expressed as gallic acid) and five benzophenone derivatives identified tentatively as galloylated maclurin and iriflophenone glucosides, and 21 (15.5 mg/g DM) and eight gallotannins (0.2 mg/g DM) found in the seed and pulp, respectively. Among the gallotannins, some identified derivative compounds were assigned provisionally as iso-penta-*O*-galloyl-glucose, iso-hexa-*O*-galloy-glucose, penta-*O*-galloyl-glucose, tetra-*O*-galloyl-glucose, and hexa-*O*-galloy glucose (Berardini et al., 2003). Some identified derivatives of quercetin were consigned as quercetin-3-*O*-galactoside and quercetin-3-*O*-glucoside (Ramirez et al., 2013), quercetin-3-*O*-xyloside, quercetin-3-*O*-arabinopyranoside, and quercetin-3-*O*-arabinofuranoside (Schieber et al., 2003).

Ramirez et al. (2013) reported that the peel of Pica cultivar from Chile presented the highest content of total phenolic compounds (66.02 mg/100 g FW) analyzed by HPLC–PDA, and these authors detected 18 compounds present in Pica pulp and 13 in Pica peel more than what was detected in the peel and pulp of Tommy Atkins from Chile. The phenolic compounds identified in these fruits were three procyanidin dimers, seven phenolic acid derivatives, and four xanthones including homomangiferina, mangiferin, and mangiferin gallate in both peel and pulp of Pica and Tommy Atkins cultivars; only dimethyl mangiferin was identified in Tommy pulp (Ramírez et al., 2013).

### Flavonoids and Other Polyphenolic Compounds

Polyphenols are a class of phytochemicals abundant throughout the plant kingdom. These molecules are generally involved in protecting plants from the ultraviolet radiation, aggression by pathogens, and reactive oxygen species (ROS) (Manach et al., 2004; Manach et al., 2005; Matheyambath et al., 2016). The most abundantly occurring polyphenols in plants are flavonoids, stilbenes, and lignans, of which flavonoids account for 60% of dietary polyphenols (Ramos, 2007; Van Breda et al., 2008).

Current interests are the antioxidant, anti-inflammatory, and anticarcinogenic activities of polyphenolic phytochemicals. The relevant polyphenols in the mango fruit related with the antioxidant capacity and/or quantity are the class of flavonoids (catechins, quercetin, kaempferol, rhamnetin, anthocyanins, and tannic acid) and the class of xanthones: mangiferin (Manach et al., 2004; Masibo and Qian, 2008). In the pulp of mango, the major flavonols are glycosides of quercetin (glucose, galactose, rhamnose, xylose, and arabinose), whereas kaempferol, isorhamnetin, fisetin, and myricetin are present in minor levels (Berardini et al., 2003; Ribeiro et al., 2008; Ramirez et al., 2013; USDA, 2018).

The USDA Nutrient Data Laboratory Flavonoid Database (https://www.ars.usda.gov/northeast-area/beltsville-md-bhnrc/beltsville-human-nutrition-research-center/nutrient-data-laboratory/) includes the data for 500 food items and 28 relevant monomeric dietary flavonoids (flavonols, flavones, flavanones, flavan-3-ols, and anthocyanidins). The USDA, through this flavonoid database, has reported that 100 g of edible portion of mango fruit contains anthocyanidins (cyanidin, 0.10 mg; delphinidin 0.02 mg; and pelargonidin, 0.02 mg), the flavan-3-ol (+)-catechin (1.72 mg), traces of the flavones apigenin (0.01 mg) and luteolin (0.02), the flavonols kaempferol (0.05 mg) and myricetin (0.06 mg) (Haytowitz et al., 2018). In addition, the Nutrient Database of USDA has reported that mango fruit (Tommy Atkins, Kent, Keitt, and Haden) contains isoflavones (0.01 mg), proanthocyanidins dimers (1.8 mg), trimers (1.4), and four to six dimers (7.2 mg). Thus, the main flavonoids that have detected in mango flesh are quercetin and glycosides derivatives; the most relevant is the flavonol glycoside quercetin 3-galactoside (22.1 mg/kg), followed by quercetin 3-glucoside (16.0 mg/kg), quercetin 3-arabinoside (5.0 mg/kg), and quercetin aglycone (3.5 mg/kg) (Ediriweera et al., 2017; Matheyambath et al., 2016). Some mango cultivars were grown in Thailand (Tommy Atkins, Mani, Ngowe, R2E2, Kent, Jose, Mini-mango, Haden, Heidi, and Kaew Mon Duen Gao) has been found to contain glycosides of quercetin between 3.5 and 1,309.1 mg/100 g fruit (diglycoside, 3-*O*-gal, 3-*O*-glc, 3-*O*-xyl, 3-*O*-arap, 3-*O*-araf, and 3-*O*-rha), kaempferol 3-glc (6.7–77.3 mg), rhamnetin-3-*O*-gal/glc (5.4–734.4 mg), and quercetin (1.7–19.3 mg) (Berardini et al., 2003).

The seed and peel of mango fruit are also considered promising sources of polyphenols (Ribeiro et al., 2007; Ribeiro et al., 2008), with a total phenolic content for these residues of 6–8% of DM in Uba cultivar from Brazil, which is 4.6 and 7.3 times higher, respectively, than the content of the pulp, and a similar profile was reported for the flavonoids and xanthones of this variety (Ribeiro et al., 2008).

The xanthones are molecules formed by a C6–C3–C6 backbone structure with hydroxyl, methoxyl, and isoprene units linked to the A and B rings, which mostly occur as ethers or glycosides (Negi et al., 2013). Six xanthone derivatives have been identified (mangiferin, dimethyl mangiferin, homomangiferin, mangiferin gallate, isomangiferin, and isomangiferin gallate); among this group mangiferin (C2-*b*-d-glucopyranosyl-1,3,6,7-tetrahydroxyxanthone), a C-glucosyl xanthone, is broadly distributed in higher plants, with demonstrated pharmacological and antioxidant activities. Mangiferin can be obtained from the bark, fruits, roots, and leaves of *Mangifera indica* Linn (Matheyambath et al., 2016). It has also described that mangiferin is able to activate anticancer, antimicrobial, antiatherosclerotic, antiallergenic, anti-inflammatory, analgesic, and immunomodulatory activities (Berardini et al., 2003; Ribeiro et al., 2008; Saleem-Dar et al., 2016; Ediriweera et al., 2017; Imran et al., 2017).

The content of mangiferin and derivatives is higher in the peel from Pica and Tommy Atkins mango fruit (22.15 and 9.68 mg/100 g FW, respectively) than in the pulp, 4.24 and 3.25 mg/100 g FW, respectively (Ramirez et al., 2013). In Uba and Tommy Atkins cultivars from Brazil, mangiferin was detected as 12.4 and 2.9 mg/kg DM, respectively, but it was not detected in Palmer pulp (Ribeiro et al., 2008).

In another analysis of mangiferin of the pulp of 11 cultivars grown in China, only in five of them was mangiferin reported (0.032–3.20 mg/100 g FW) (Luo et al., 2012), but interestingly, in the pulp of Azúcar cultivar grown in Colombia, it was 11.5 mg/100 g FW (data not shown).

For the derivative compounds of mangiferin, some characteristic peaks were identified as corresponding to compounds identified provisionally as maclurin-mono-*O*-galloyl-glucose, maclurin-di-*O*-galloyl-glucose, iriflophenone di-*O*-galloyl-glucose (Berardini et al., 2003), and mangiferin gallate (Schieber et al., 2003).

### Pigments: Chlorophylls, Carotenoids, and Flavonoids

#### Chlorophylls

The color of the fruit peel is an important factor of mango maturation indices and quality, which changes from green to orange, yellow, or red flush, depending on the type of cultivar. In mango fruit, the green pigmentation is attributed to the presence of chlorophylls (Sudhakar et al., 2016; Nelson and Cox, 2017). Two types of chlorophyll have been detected in mango fruit, chlorophylls *a* (blue-green) and *b* (yellow-green), in a ratio of 3:1 (Medlicott et al., 1986; Saengnil and Kaewlublae, 1997; Lee and Schwartz, 2005).

In Tommy Atkins mango fruit, chloroplasts contain between 7 and 10 thylakoids per stack, connected through single intergranular thylakoids (Medlicott et al., 1985; Medlicott et al., 1986). Thylakoid system collapse inside the chloroplast at the beginning of ripening is associated with the loss of chlorophyll (Medlicott et al., 1986). Moreover, the reduction of chlorophyll levels in the peel is correlated with the increase of β-carotene content as ripening advances (Ketsa et al., 1999).

Chlorophyll and carotenoids are responsible for the color in some fruits (Sánchez-González et al., 2016). Several studies describe that chlorophyll breakdown is associated with the maturity of some fruits; (Du et al., 2014; Charoenchongsuk et al., 2015; Sánchez-González et al., 2016; Wei et al., 2019). When fruit appears green, an abundance of chlorophyll masks the carotenoids. The yellow color of carotenoids is unmasked by chlorophyll degradation during ripening (Charoenchongsuk et al., 2015). Taking this into account, the content of chlorophyll could be used as an indicator for the harvest in some fruits but not in others; for example, Charoenchongsuk et al. (2015) in their study show that ‘Le Lectier’ pears turned yellow during ripening with concomitant loss of chlorophylls *a* and *b* and carotenoids. In contrast, ‘La France’ pears stayed green even when fully ripe, because chlorophyll content did not change significantly.

Lechaudel et al. (2010) demonstrated that the greener color of the fruit is affected according to the position in the tree. The high values of chlorophyll correspond to green color of the peel and the flesh for fruits inside the canopy, whereas low values of chlorophyll are present in the fruits located in the top of the canopy.

The reduction in the chlorophyll content in the fruit is attributed to ethylene, which up-regulates the *de novo* synthesis of the enzyme chlorophyllase in the peel during ripening (Mir et al., 2001; Choo, 2018). Besides, chlorophyll can be also degraded by the peroxidase activity able to open the porphyrin ring, producing the loss of color (Kato and Shimizu, 1985). The peroxidase activity in the peel of unripe and ripe Tongdum Thai mango was lesser than that in Nam Dokmai mango, which can explain the increased content of chlorophyll in Tongdum (Ketsa et al., 1999).

#### Carotenoids

Mango fruit is rich in carotenoid compounds. These molecules are lipid-soluble stains contributing to yellow-orange colors of mango fruit and red colors when mango is ripe, although the reddish color of peel in several varieties is due to anthocyanins (Masibo and Qian, 2008; Sivankalyani et al., 2016). Carotenoids are located in the chromoplasts, often masked by chlorophyll and non-photosynthetic plant tissues (Tanaka et al., 2008; Choo, 2018). In the chloroplasts, carotenoids act as an accessory pigment in light harvesting and as antioxidants converting the triplet chlorophyll to the singlet ground state (Arafat, 2005; Alcaíno et al., 2016). These compounds are classified in carotenes (α-carotene, β-carotene and γ-carotene), and xanthophylls (auroxanthin, antheraxanthin, neoxanthin, lutein, violaxanthin, and zeaxanthin) (Cano and Ancos, 1994; Varakumar et al., 2011; Eskin and Hoehn, 2013).

Sixteen carotenoids have been identified in fully ripe mango fruit, of which β-carotene (all-trans) account for 60% of the total carotenoids in the fruit (Saleem-Dar et al., 2016), while at the green stage, lutein (9- or 9′-*cis*-lutein) is the most representative, followed by many xanthophylls during the early ripening stage and during late ripening phases (Bramley, 2013; Ediriweera et al., 2017).

The carotenoid levels in fruit are directly affected by the development and the environmental conditions during fruit growth (Bramley, 2013). The variation in total carotenoid content in four different varieties of mango ripe and unripe was analyzed by Ellong et al. (2015). They reported that total carotenoids of mango cultivars Bassignac, Green, Julie, and Moussache were between 276.17 (Green) and 2,183 μg (Bassignac) per 100 g FW in the unripe stage. These values increased at the ripe stage, between 603.35 (Moussache) and 4,138.50 μg (Bassignac) of total carotenoids per 100 g FW (Ellong et al., 2015). A similar profile was observed in the total carotenoid contents of 12 mango varieties from Bangladesh at three stages of development: green (0.003 mg/100 g of pulp), semi-ripe (0.07 mg/100 g of pulp), and ripe (0.25 mg/100 g of pulp), indicating that the carotenoid content increased from green into ripe stage (Haque et al., 2015). Another study also compared the total carotenoid levels in the edible portion of the mango, variety Azúcar, at green, semi-ripe, and ripe stages (11.1, 11.8, and 10.7 mg of total carotenoids/100 g FW of edible portion, respectively). A significant difference only was found between the ripe and semi-ripe stages (Corrales-Bernal et al., 2014). The total carotenoid contents of the variety Azúcar were similar to those reported for the cultivars Keitt and Kent (10.4 and 12.9 mg/100 g pulp, respectively); higher than those of Tommy Atkins, Tainong No. 1, Irwin, and JinHwang cultivars (4.9, 5.2, 3.7, and 2.6, respectively) (Liu et al., 2013); but lower than those of Ataulfo mango (26.1 mg) (Manthey and Perkins-Veazie, 2009) at the ripe stage.

The total carotenoid content was also analyzed for the cultivars Haden, Tommy Atkins, Ubá, and Palmer from Brazil; this content ranged from 1.91 mg/100 g (Haden) to 2.63 mg/100 g (Palmer). The total carotenoid, β-carotene, accounted for 661.27 (Palmer) to 2.220 µg/100 g (Ubá) (Ribeiro et al., 2007). These values are higher than those in the Indian cultivars (Saleem-Dar et al., 2016). Comparable results were reported by the Nutrient Database of USDA (2018) for β-carotene (640 µg/100 g), α-carotene (9 µg/100 g), β-cryptoxanthin (10 µg/100 g), lutein and zeaxanthin (23 µg/100 g), and lycopene (3 µg/100 g) in Tommy Atkins, Kent, Keitt, and Haden mangoes.

Although the differences in the composition of carotenoids between different varieties may be due to environmental and genetic factors, state of maturation, production, and postharvest handling techniques, they can also be attributed to the analytical methods employed and to the unstable nature of carotenoids (Burton-Freeman et al., 2017). More than 25 carotenoids (free form, butyrates, and esterified), have been identified, but the major carotenoids in mango flesh seem to be all-*trans*-β-carotene, and all-*trans*- and 9-*cis*-violaxanthin (Mercadante et al., 1997; Ornelas-Paz et al., 2007; Petry and Mercadante, 2016).

The analysis of seven ripe mango cultivars (Ataulfo, Manila, Criollo, Paraíso, Haden, Kent, and Tommy Atkins) by HPLC coupled to a C30 stationary phase and diode array, fluorescence, and mass detectors showed that the highest content of carotenoid was contributed by all-*trans*-β-carotene (0.4 and 2.8 mg/100 g FW), with mangoes Haden and Ataulfo having the highest concentration, followed by all-*trans*-violaxanthin (0.5–2.8 mg/100 g FW) and 9-*cis*-violaxanthin (0.4–2.0 mg/100 g FW) (Ornelas-Paz et al., 2007). In a mango cultivar from Brazil, it was identified by HPLC and MS that all-*trans*-violaxanthin (2.1 mg/100 g FW) was the major carotenoid, followed by all-*trans*-β-carotene (1.5 mg/100 g FW) and 9-*cis*-violaxanthin (1.0 mg/100 g FW) (Mercadante et al., 1997).

A study of five mango varieties harvested at different times [Tommy Atkins (México, Brazil, and Ecuador), Kent and Keitt (Mexico), and Haden (Peru)] was performed to compare the β-carotene content over 1 year of harvest. This study showed that the concentration of β-carotene ranged from 5 (Tommy Atkins) to 30 mg/kg FW (Ataulfo) among the five varieties (Manthey and Perkins-Veazie, 2009), indicating that the type of cultivar had a greater influence on the levels of β-carotene but not the country of origin or harvest date (Manthey and Perkins-Veazie, 2009).

### Volatile Compounds

The volatile compounds of mango are characterized by having low molecular weight (<400 Da), and in a wide range of functional groups, they can be in free or glycoside form. Also, they have a high vapor pressure that allows them to disperse easily and quickly in air, water, and soil (Sharifi and Ryu, 2018). This group of compounds that determine the characteristic aroma of the fruit is commonly present in small quantities, approximately 50 ppm or less, which comprises mixtures of monoterpenes, sesquiterpenes, and volatile oxygenates (monoterpenes, sesquiterpenes, esters, lactones, alcohols, aldehydes, ketones, volatile fatty acids, some degradation product of phenols, and some carotenoids) (Bender et al., 2000; Pino et al., 2005; Lebrun et al., 2008; Pandit et al., 2010; Li et al., 2017). They have a heterogeneous distribution quantitatively and qualitatively among cultivars, maturity stage, and tissues of the fruit (MacLeod and Pieris, 1984; Adedeji et al., 1992; Andrade et al., 2000; Lalel et al., 2003a; Lalel et al., 2003b; Lalel et al., 2003c; Estarrón and Martindel Campo, 2016). Singh et al., (2004) analyzed more than 285 different volatile compounds in mango fruit including 7 acids, 55 alcohols, 31 aldehydes, 26 ketones, 14 lactones, 74 esters, 69 terpene hydrocarbons, and 9 other compounds (Singh et al., 2004). The most abundant volatile components in mango germplasms from China, the Americas, Thailand, India, Cuba, Indonesia, and the Philippines are monoterpenes (Li et al., 2017).

The content of these molecules can vary according to the method used for their extraction and identification and quantification, such as solid-phase microextraction (SPME) (Lalel et al., 2003a; Lalel et al., 2003b; Lalel et al., 2003c; Rivera et al., 2017), liquid–liquid extraction (Ollé et al., 1997), simultaneous distillation–extraction (MacLeod and Pieris, 1984; Pino et al., 1989; Andrade et al., 2000; Pino et al., 2005), static or dynamic headspace (Malundo et al., 1997), and solid-phase extraction (Adedeji et al., 1992).

Pino et al., (2005) analyzed the volatile compounds of 20 mango cultivars from the National Botanic Garden in Havana, Cuba, using simultaneously distillation–extraction, gas chromatography (GC), and GC coupled to MS (GC–MS). They reported 180 volatile compounds from 372 identified with a concentration ranging from 18 to 123 mg/kg of fresh fruit (Pino et al., 2005). In these cultivars, the major volatile compounds were terpene hydrocarbons such as delta-3-carene, a dominant compound present in other mango cultivar grown in Venezuela extracted by similar methods (MacLeod and Gonzalez de Troconis, 1982); followed by limonene also present in cultivars Baladi (Ollé et al., 1998), Carlota, and Bacuri (Andrade et al., 2000); terpinolene characteristics of Sri Lanka (MacLeod and Pieris, 1984); and cultivars grown in Australia (Bartley, 1988). The following quantitative important class of volatile compounds in mango grown in Cuba was 90 aliphatic, 16 aromatics, and 8 terpene esters found, with ethyl acetate and ethyl butanoate as the major esters; lactones were also detected (Pino et al., 2005; Desphande et al., 2016).

Some other examples of volatile constituents detected in the pulp of Tommy Atkins mangoes are pinene, limonene, α-terpinolene, d-carvone, β-elemene, α-bourbonene, β-cubebene, α-cubebene, aromadendrene, α-humulene, germacrene D, and *cis*-caryophyllene (Rivera et al., 2017). Some of the compounds found in the variety Bowen grown in Australia include ethyl butanoate, thujene, ethyl decanoate, β-caryophyllene, 3-carene, myrcene, ethyl butenaonate, and β-phellandrene (Correa, 2012). Some of the compounds found in the sap of seven mango varieties grown in India include β-myrcene, *trans*-/*cis*-ocimene, and limonene (Johna et al., 1999).

Some of the compounds detected in the mango sap from nine cultivars grown in Pakistan included (+)-3-carene, sabinene, α-phellandrene, α-humulene, γ-terpinene, α-pinene, and (−)-*trans*-caryophyllene (Musharraf et al., 2016). The 3-carene is reported as the most abundant for the varieties Bizcochuelo, S. Hayden, Tommy Atkins, Kent, Keitt, M. Bingué, Tête de chat, Palmer, and Irwin (Torres et al., 2012; Liu et al., 2013). In the varieties Bizcochuelo, S. Hayden, and Amelie, the caryophyllene is abundant (Pino et al., 1989), while α-terpinolene, a representative volatile compound, is in the cultivars Palmer, Kensington Pride, Tainong No. 1, JinHwang, and Keitt (Liu et al., 2013).

Volatiles in Tommy Atkins mangoes grown in Mexico were identified using SPME and GC–MS, which included pinene, limonene, α-terpinolene, d-carvone, β-elemene, α-bourbonene, β-cubebene, α-cubebene, aromadendrene, α-humulene, germacrene D, and *cis*-caryophyllene (Rivera et al., 2017). It has also been observed that mango fruit grown in the American continent commonly contains terpinene, sabinene, α-copaene, ethyl dodecanoate, and benzaldehyde, whereas the following terpenes were found in all the varieties studied: limonene, α-pinene, β-pinene, 3-carene, α-terpinolene, and α-humulene (Pino et al., 2005; Correa 2012; Estarrón and Martindel Campo, 2016).

## Changes in Nutritional and Phytochemical Components During Fruit Development, Ripening, and Senescence

The beneficial effects of the mango components in human health due to their dietary, nutritional, and biological properties are affected by fruit development, ripening, and senescence. The maturity stage is a significant aspect that affects the compositional quality of fruit including nutritional factors, since during fruit ripening occur important biochemical, physiological, and structural changes.

The development of mango fruit occurs in four phases: 1) the juvenile until 21 days from fruit setting, when a rapid cellular growth occurs; 2) phase of maximum growth between 21 and 49 days from fruit setting, involving cell enlargement and initiation of maturation; 3) maturation and ripening stage between 49 and 77 days from fruit set, when the respiration climacteric and ripening process occur; and 4) senescence stage from day 77 from fruit set onwards, considered as the post-ripening stage, which is susceptible to microbial attack followed by decay and death (Tharanathan et al., 2006).

**Figure 2:**
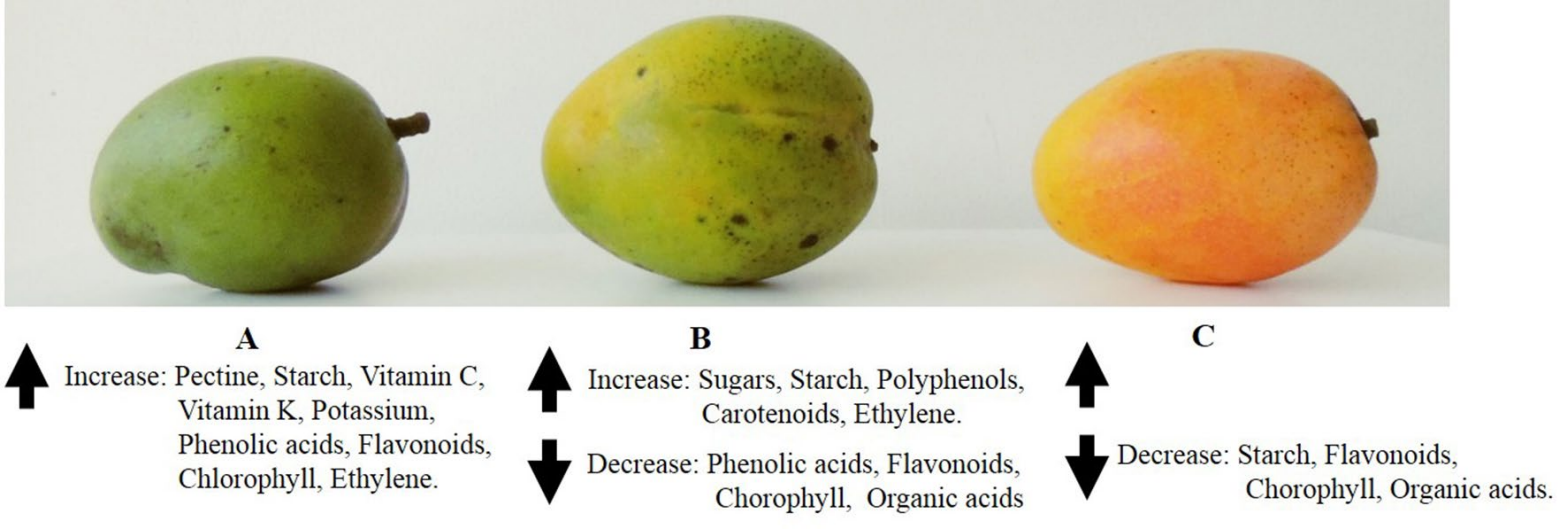
Pictorial representation of ripening stages of “Criollo” mango fruit where changes of most representative phytochemicals are indicated.

During the growth and ripening processes of mango fruit, changes in chemical composition occur (**Figure 2**), including decrease in ash level with some rise when nearing maturity, fiber remaining more or less constant, increase in the content of alcohol-insoluble solids due to starch accumulation, change of structural polysaccharides, and hydrolysis of starch into sugars, followed by fruit softening, biosynthesis of volatile compounds, chloroplast degradation, and chromoplast and carotenoid biosynthesis (Joas et al., 2012; Wongmetha et al., 2015; Matheyambath et al., 2016; Saleem-Dar et al., 2016). All these changes are the consequence of physiological and biochemical events controlled during ripening of fruit involving the fruit softening that affects the eating quality (Gill et al., 2017).

The increased activity of some enzymes through ripening may favor internal physiological disorders referred to as the internal flesh breakdowns such as a soft nose, jelly seed, and spongy tissue (Wainwright and Burbage, 1989). The occurrence and intensity of the alterations mentioned above depend on climate, geographical localization, and varieties; and the symptoms exhibit at the final step of fruit growth and maturation (Thomas et al., 1993; de Oliveira Lima et al., 1999). Biochemical studies showed increasing activity of the enzyme amylase through fruit growth, and this activity decreases towards the maturity stage (Sen et al., 1985).

It is important to note that depending on the variety of mango, the maturity process occurs starting in the skin towards the seed, as in the case of the Alphonso variety, while other varieties mature in the opposite direction such as in Haden, Kent, and Tommy Atkins.

The development of the fruit and the maturation process entail changes at the transcriptional level as demonstrated in the Alphonso variety, where it was observed that 4,611 transcripts correspond to different enzymes (oxidoreductase, transferase, hydrolase, ligase, lyase, and isomerase) and in which transferase enzymes were abundant followed by hydrolases in the tissues analyzed: flower, whole fruit 30 and 60 days after pollination, mature raw fruit, and pulp (green, mid-ripe, and ripe fruit) (Deshpande et al., 2017). This study identified genes encoding enzymes involved in 142 metabolic pathways of primary and secondary metabolites (Deshpande et al., 2017). Novel transcripts involved in the biosynthesis of monoterpenes, sesquiterpenes, diterpenes, lactones, and furanones related with flavor have been also identified in Ataulfo mangoes, which are differentially regulated during fruit development; in addition, 79 novel transcripts of inhibitors of cell wall-modifying enzymes were also identified, which suggests that the activity of enzymes can be controlled (Deshpande et al., 2017).

Starch exhibits a rapid rate of accumulation at the beginning of fruit development and decreases later, but it continues increasing until maturity (Tharanathan et al., 2006). For example, Alphonso mango shows a rise from 1% to 14% in starch content throughout development (Quintana et al., 1984). A similar profile was observed in JinHwang mango grown in Taiwan (Wongmetha et al., 2015). At the end of the maturity stage, reducing and non-reducing sugars are found to be increasing (Mann et al., 1974). During Tommy Atkins mango ripening, starch is hydrolyzed during the first week of harvest, but as the fruit becomes over-ripe, the content of starch and amylase activity is substantially reduced (de Oliveira Lima et al., 2001).

Several studies showed many compositional and metabolic differences between the healthy and damaged tissues. de Oliveira Lima et al. showed that sugars in the spongy pulp tissue (disease of fruit) are a consequence of the presence of starch that has not been hydrolyzed because of low activity of amylase enzyme during ripening in this disorder (de Oliveira Lima et al., 2001).

The last ones are responsible for the hydrolysis of sucrose. The accumulation of sucrose in Irwin mango was associated with the decrease of sucrose phosphate synthase activity, whereas the activities of acid invertase, neutral invertase, and sucrose synthase decreased at five developmental stages (50, 70, 90, 110, and 130 days after anthesis). A similar result was observed in JinHwang mango 110 days after anthesis (Wongmetha et al., 2015); moreover, the acid invertase activity was the dominant enzyme in sugar accumulation and quality of mango fruit, which propose that mango fruit of Irwin and JinHwang cultivars should be harvested after physiological maturity at 110 days after anthesis. During the initial stages of fruit development, sucrose phosphate synthase activity was higher than sucrose synthase activity and then decreased during the advances of the development of the fruit; in contrast, sucrose synthase activity increased from 0.27 U sucrose/min/g FW at 50 days after anthesis to 2.32 U sucrose/min/g FW at 130 days after anthesis (Wongmetha et al., 2015). During ripening, sucrose increased from 5.8% to 14.2% FW, while the pH changed from 3.0 to 5.2, whereas in the post-climacteric stage, the total acidity ranged from 0.13% to 0.71%, and the level of non-reducing sugars diminishes to 0.6% (Tharanathan et al., 2006).

Pectins are responsible for fruit texture. Pectin rises in the fifth week of mango fruit setting until the stone is formed, and then the pectin content decreases, leading to the fruit softening because of enzymatic degradation and solubilization of protopectin (Jain, 1961). Mango fruit pulp is composed of parenchymatous tissues that consist of calcium salts of pectin located in the cell wall during the early stages of cell growth (Voragen et al., 1995). The deesterification of pectins and losses of calcium ions are characteristic of ripening fruit because of cell wall breakdown and dissolution of middle lamella (Tharanathan et al., 2006). When mango cell walls are degraded during ripening, monosaccharides of the pectin complex are released, and the resulting water-soluble pectic materials in the cell walls lose arabinose and galactose accounting for the galacturonan-rich polysaccharides in the mesocarp (Lizada, 1993).

Moisture content was observed to range from 780.78 to 790.78 g/kg FW in green Tommy-Kent and Tommy Atkins mangoes and increased significantly in the mature stage (835.55 to 849.04 g/kg FW), which can be attributed to the catabolism or modifications of cell wall fibers and, therefore, to water liberation (Barbosa Gámez et al., 2017).

A reduction in the soluble protein content happens up to 44 days after fruit setting and rises until 96 days (Lakshminarayana et al., 1970). At maturity, the amino acids alanine–arginine, glycine–serine, and leucine–isoleucine are predominant but decrease during ripening, with the exception of alanine (Lakshminarayana et al., 1970). Barbosa Gámez et al. (2017) reported that protein content in immature Tommy-Kent and Tommy Atkins mangoes was 40.45 and 37.02 g/kg FW, respectively, higher than in mature stage (12.10 and 13.87 g/kg FW, respectively); this is because there is an increase of metabolic enzymes required in the maturation and ripening process, while at the senescence stage, enzymes decrease (Andrade et al., 2012; Barbosa Gámez et al., 2017).

With respect to fatty acid content during mango ripening, it has been reported that there is an increase in triglyceride content in Alphonso mango fruit pulp, followed by modifications in the composition of fatty acid of the pulp (Bandyopadhyay and Gholap, 1973). Similar patterns have been also observed in mango cultivars from India (Selvaraj et al., 1989). During the ripening, an equal distribution of palmitic acid and palmitoleic acid it has observed, as well as a reduction in linoleic acid content and an increase in linolenic acid content (Desphande et al., 2016). The palmitic acid is incorporated to hydroxy fatty acids that are precursors of lactones; thus, a correlation between the aroma and flavor was observed using the ratio of palmitic acid to palmitoleic acid (Bandyopadhyay and Gholap, 1973; Lizada, 1993). During the preclimateric and climateric stages, the mitochondria increase its capacity to oxidize fatty acids such as stearic and oleic acids, producing precursors for the synthesis of carotenoids and terpenoid volatiles (Lizada, 1993).

Organic acid content decreases as the mango fruit ripens, and consequently, the titratable acidity declines (Shashirekha and Patwardhan, 1976). It has been reported that the enzyme activities involved in the Krebs cycle of mango fruit change during ripening (Baqui et al., 1974; Lizada, 1993). For example, the citrate synthase activity is drastically decreased, the activities of isocitrate and succinate dehydrogenases increase (Lizada, 1993), and malic enzyme in mango pulp presents the highest activity just before the climacteric peak (Dubery et al., 1984). Citrate synthase activity decreases during ripening, while isocitrate dehydrogenase and succinate dehydrogenase activities increase (Baqui et al., 1974).

Vitamin C contents in the pulp decrease during mango fruit ripening and are maximum in the early stages of growth (Robles-Sanchez et al., 2009); in spite of that, the ripe mango is an important source of vitamin C (Matheyambath et al., 2016). In addition, the amount of loss varied by species (Hu et al., 2018). One of the first studies on vitamin C developmental patterns in different mango cultivars (Amini, Mullgoa, Pico, and Turpentine) reported a reduction in the levels of the flesh 5 weeks after fruit setting until maturity, and smaller fruits with average age of about 5 weeks had an average vitamin C value of 88 mg/100 g of flesh mango fresh, but at maturity (16 weeks), these same changes were around only 22 mg/100 g (Spencer et al., 1956). A similar pattern has been reported for Tommy Atkins, Keitt, and Kent mangoes (Manthey and Perkins-Veazie, 2009). Moreover, Keitt mango showed significantly higher vitamin C content than did the other two varieties in the ripe phases, because of its direct inhibition of polyphenol oxidase (PPO) that probably confers better color and flavor retention during handling and processing (Robinson et al., 1993; Palma-Orozco et al., 2014). This reduction in vitamin C levels has been also explained based on the coenzyme function for the ACC-oxidase involved for ethylene synthesis, or as a substrate for the oxalate and tartrate biosynthesis (Mazid et al., 2011). In spite of this, the consumption of 300 g of mature Kent and Tommy Atkins mangoes contains 645 and 1,410 mg vitamin C/kg FW, respectively, which exceeds the dietary recommendations of 75–90 mg/day (WHO, 2003; Ibarra-Garza et al., 2015; Barbosa Gámez et al., 2017).

The content of vitamin E also changes through the ripening of mango fruit. Barbosa Gámez et al. (2017) reported that the content of this vitamin is highest at the immature stage (cv. Tommy-Atkins 91 mg/kg FW); however, when the fruit is ripened, the values were lower (65.81 mg/kg FW). On the other hand, Tommy-Kent mango presented an increase in vitamin E from the immature to mature stage (51.96 to 77.50 mg/kg).

The content of the group of B vitamins changed significantly at the mature stage compared with the immature stage (Barbosa Gámez et al., 2017). Niacin increased by 3.0- and 1.75-folds, pyridone increased by 2.8- and 3.4-folds, riboflavin increased by 3.1- and 2.9-folds, and thiamine increased by 2.2- and 2.5-folds in mature Tommy-Kent and Tommy Atkins mangoes, respectively (Barbosa Gámez et al., 2017).

The content of phenolic compounds (polyphenols and phenolic acids) changes during development until maturity due to their capacity for neutralizing free radicals, which are naturally produced during ripening processes or senescence (Palafox-Carlos et al., 2012a; Palafox-Carlos et al., 2012b). Total polyphenol contents in the immature stage in Kent and Tommy Atkins mango fruit were 4.81 and 4.42 g/kg, respectively, while in the mature stage, they were 5.24 and 4.18 g/kg, respectively (Barbosa Gámez et al., 2017). Flavonoids were low during mango ripening because of low expression of flavonol synthase as observed in Ataulfo mango Palafox-Carlos et al., 2012a; Palafox-Carlos et al., 2012b). A similar profile was observed in Keitt and Xiangya mangoes grown in China for the content of total polyphenols in the pulp and peel, where a decrease was observed during ripening [in Keitt, 97.59 to 59.43 and 1,207.02 to 641.90 mg gallic acid equivalent (GAE)/100 g FW; in Xiangya, 96.15 to 45.15 and 723.67 to 358.62 mg GAE/100 g FW], and flavonoids in green mango were 45% more than those in mature mango (Hu et al., 2018).

Several phenolic compounds have been identified in four ripening stages selected according to the percentage of yellow color in the peel of Ataulfo mango, including gallic, chlorogenic, protocatechuic, and vanillic acids (Palafox-Carlos et al., 2012a; Palafox-Carlos et al., 2012b). Chlorogenic acid in Ataulfo mango showed a content of 28 mg/100 g DW in the ripening stage 1 (0–10% yellow surface) and increased to 301 mg/100 g DW in the ripening stage 4 (71–100% yellow surface), whereas gallic acid content in the first ripening stage (0–10% yellow surface) was 94.6 mg/100 g DW, without significant differences with respect to ripening stages 2 and 3 (11–40% and 41–70%, yellow surface, respectively), but in ripening stage 4, it reached 98.7 mg/100 g DW. A similar profile was reported for vanillic acid (16.9 mg/100 g DW) in the first ripening stage to 24.4 mg/100 g DW in ripening stage 4; and the protocatechuic acid had a concentration of 0.48 mg/100 g DW in the first ripening stage and increased to 1.1 mg/100 g DW in the last ripening stage (Palafox-Carlos et al., 2012a; Palafox-Carlos et al., 2012b).

The pigmentation of the pulp in mango fruit occurs from the seed outwards (Tharanathan et al., 2006). The chlorophyll present in the unripe stage is degraded during mango ripening, and earlier present pigments and biosynthesis of anthocyanins and carotenoids are uncovered (Medlicott et al., 1986; Lizada, 1993; Tharanathan et al., 2006). Mature green mango contains three times more chlorophyll and marginally more β-carotene in the peel, where the enzyme activities of chlorophyllase and peroxidase are about half those of the ripe yellow fruit (Medlicott et al., 1986; Selvaraj and Kumar, 1994; Ketsa et al., 1999). The activity of these enzymes leads to a complete reduction of peel chlorophyll *a* during ripening from 2.2 in unripe fruit to 0.8 µg/cm^2^ in ripe fruit (Medlicott et al., 1985; Medlicott et al., 1986).

On the other hand, the total carotenoid content of the peel increases approximately fivefold during ripening, probably due to carotenoid synthesis, in addition to underlying pulp carotenoids (Medlicott et al., 1986). The increase in total carotenoids during maturity has been considered as ripening index and harvest indicator; that is, the cultivars Sensation and Xiangya grown in China showed significantly higher total carotenoid content values in peels than in pulp; on the contrary, the variety Keitt also grown in China exhibited an adverse result (Hu et al., 2018). Ajila et al. (2007) reported that total carotenoid content was fourfold to eightfold greater in ripe mango peel than in unripe mango peel, whereas the research of Hu et al. (2018) showed around three to eight times higher content in ripe mango peel (Hu et al., 2018).

The accumulation of anthocyanins, which contribute to the red coloration of the mango peel, is dependent on the level of sun exposure (Medlicott and Thompson, 1985). For example, Saengnil and co-workers showed that mangoes (cv. Kent) that were covered with brown paper bags had lower levels of anthocyanins and less redness in the peel than were mangoes not covered (Saengnil and Kaewlublae, 1997). The accumulation of anthocyanins and flavonoids in the peel of mango fruit protects against the chilling injury and pathogen infection, probably because of the antioxidant capacity of these polyphenols inhibiting lipid peroxidation and reduction of decay incidence (Sivankalyani et al., 2016). These findings may lead to new strategies as selection of the resistant red fruit or technical methodologies that would increase the content of anthocyanins and flavonoids in mango fruit peel for improving postharvest traits and for improving diminishing losses (Sivankalyani et al., 2016).

The synthesis of a mixture of volatile compounds is associated with the flavor and aroma during fruit ripening as mentioned in a previous section. This is a consequence of ethylene production as volatile components accumulate from skin and pulp, improving fruit aroma and flavor (Lizada, 1993; Sergent et al., 1993; Singh and Janes, 2001; Tharanathan et al., 2006). The skin of Alphonso mango has an important terpene content, while the pulp is rich in lactone (Chidley et al., 2013). A similar profile of the volatile composition of skin and pulp of green Khieosawoei mango grown in Thailand (Tamura et al., 2001) and Kensington Pride mangoes (Lalel et al., 2003a; Lalel et al., 2003b; Lalel et al., 2003c) was observed. The major volatile components present in ripe mango fruit are terpenes, while some other hydrocarbons, esters, and alcohols have also been detected in this stage (Hunter et al., 1974, Pino et al., 1989).

## Changes in Nutritional and Phytochemical Components During Postharvest Handling Practices

After harvest of mango fruit, losses in quantity and quality occur, affecting the content of nutritional and phytochemical components at different points in the handling chain (Esguerra and Rolle, 2018). This is very important because consumers are interested not only in visual quality but also in health components and fruit safety (Esguerra and Rolle, 2018). There is limited information about how postharvest handling practices influence nutrient and phytochemical levels in mango fruit.

Postharvest activities include all those that are carried out with the fresh product, which can be done in the field after harvest, in collection centers, packing plant, during transport, during storage, or during marketing (Esguerra and Rolle, 2018). Some of the postharvest practices used for mango include i) trimming, ii) delatexing/desapping, iii) sorting/grading, iv) disease control, and v) procedures for extending shelf life of mangoes, such as packaging and transport (Tharanathan et al., 2006; Yahia, 2011).

Trimming is the cutting of stem resulting in latex or sap stains deposited on the fruit surface, since the sap stored in the fruit ducts is under significant pressure, and after pedicel abscission, the sap falls on the peel of mango fruit (Loveys et al., 1992; Esguerra and Rolle, 2018). The main cause of mango sap burn is attributed to a deposit of volatile compounds as terpinolene and car-3-ene through the lenticels, producing a tissue damage and the enzymatic browning (Loveys et al., 1992). The development of these pigmented lesions is a response associated with stress indicators and the successive release of PPO (Dixon and Paiva, 1995) that results in membrane damage with liberation of phenolic compounds deposited into the cell wall (Beckman, 2000; Bezuidenhout et al., 2005; Du Plooy et al., 2009).

The contact of sap or latex with the skin of mango induces lenticel discoloration, a red pigmentation described as red spots on the fruit surface caused by the synthesis of anthocyanins (Kangatharalingam et al., 2002), flavonoids (Dixon and Paiva, 1995) and phenylpropanoid derivatives (Du Plooy et al., 2009). However, this lenticel spotting can be induced at storage temperatures below 10–12°C, accelerating the occurrence of red spots related with chilling injury (Pesis et al., 2000).

Prasad and Sharma (2018) compared the effect of manual and mechanical harvesting practices on total soluble solids (TSS), total carotenoids, and antioxidant activity in four Indian commercial mango cultivars grown in India. Amrapali, Chausa, Dushehari, and Langra were stored at room temperature [25 ± 4°C and 65 ± 5% relative humidity (RH)]. Prasad and Sharma (2018) reported that independent of storage days and cultivar, no significant difference was observed in TSS between the manually harvested [20.4°B (Brix)] and mechanically harvested (20.6°B) fruit. Amrapali mangoes had the highest TSS when harvested manually and mechanically (22.1°B and 22.6°B, respectively). These differing results might be due to the fact that mechanically harvested fruit continues to ripen after harvesting, allowing for high accumulation of TSS, while manually harvested fruit does not ripen post-harvesting (Pacheco et al., 2017). In relation to total carotenoid content, Prasad and Sharma (2018) reported that independent of storage days and varieties analyzed, an overall slight increase was observed in total carotenoid content when fruits were mechanically harvested (5.4 mg/100 g FW) compared with the mangoes harvested manually (5.3 mg/100 g FW). This slight increase could be due to the effect of mechanical harvesting technique on proper attainment of ripening throughout the shelf life of mango fruit (Pacheco et al., 2017), but also subjected to varietal morphology of mango fruit (Abu-Gaukh and Mohamed, 2004). However, significant differences were found for total antioxidant activity in both manually and mechanically harvested mangoes. A slight increase of antioxidant activity was observed in fruit harvested mechanically (3.00 μmol Trolox eq/g FW) than that of manually harvested fruit (2.98 μmol Trolox eq/g FW). This difference was even more obvious at the ninth day of storage, as fruit harvested mechanically had an antioxidant activity of 4.29 μmol Trolox eq/g FW compared with 4.23 μmol Trolox eq/g FW in manually harvested fruit. These results were attributed to the extent of physical abrasion, a physical damage that directly affects antioxidants such as vitamin C present inside the fruit, because this component is used up by the fruit for combating external stresses (Pacheco et al., 2017; Prasad and Sharma, 2018).

Postharvest disease control measures such as for Anthracnose are an important aspect of postharvest practices that affect the content of mango phytochemical components (Rodríguez et al., 2009; Kamle et al., 2013). At the green stage, anthracnose cannot be perceived, and the symptoms of infection are obvious when the mango ripens; this disease is caused by the *Colletotrichum gloeosporioides* (Penz) fungi that produce the enzymes polygalacturonase and pectolyase, able to degrade the cell wall (Rodríguez et al., 2009; Kamle et al., 2013), favoring the oxidation of phenol compounds catalyzed by the enzyme PPO-producing quinones that are polymerized and form the characteristic brown spots. On the contrary, when the tissues of the mango fruit are healthy and intact, the PPO enzyme is located in chloroplasts and the phenolic compounds in vacuoles, both separated, and thus, the reaction is avoided (Ploetz et al., 1994; Pérez-Márquez et al., 2016).

Pérez-Márquez et al. (2016) analyzed the content of total phenols in ‘Super Haden’ mangos grown in Cuba that are damaged and treated after harvest, to establish their relationship with the defense mechanisms against the infection. In the skin of Super Haden mangoes affected by anthracnose, a reduction in the total phenol content levels was observed (59.25, 58.63, and 56.52 mg GAE/g FW) at different degrees of infection (mild, moderate, and severe damage, respectively), whereas the total phenol content in healthy fruit was 60.5 mg GAE/g FW (Pérez-Márquez et al., 2016).

Postharvest disease control is carried out by hydrothermal and/or chemical methods (López and Castaño, 2010). Pérez-Márquez et al. (2016) compared a treatment using hot water (53°C for 5 min) with polyethylene wax [10% Total solids (ST)] and imazalil (800 mg/L), and other with polyethylene wax (10% ST) plus imazalil (800 mg/L) on Super Haden mango fruit infected with anthracnose (mild, moderate, and severe levels); they observed that the total phenol compound content of mango peel increased after treatments. On the other hand, by using hot water with polyethylene wax plus imazalil and two bags of Conserver 21 (an ethylene absorber product), the total phenol content was 37.58 and 37.11 mg GAE/g FW, respectively, compared with the control value (33.94 mg GAE/g FW). These results also indicated a correspondence with a lower disease occurrence (Pérez-Márquez et al., 2016), because this treatment disinfects the fruit by eliminating or destroying the spores or fungal mycelium (Escribano and Mitcham, 2014). Treatment with hot water also induces the expression of the protein of polygalacturonase that can inhibit fungal endopolygalacturonase, considered as an important factor for the resistance of plants to phytopathogenic fungi (Li et al., 2013).

A similar result was reported by Kim et al. (2007) in fresh, mature, green mangoes to inhibit anthracnose. Hot water treatment (46.1°C, 75 min) combined with controlled atmosphere (3 kPa of O_2_ + 97 kPa of N_2_, or 3 kPa of O_2_ + 10 kPa of CO_2_ + 87 kPa of N_2_) after 2 weeks of storage at 10°C and ripening in air at 25°C did not affect the content of gallic acid and hydrolysable tannins, while total polyphenols decreased naturally during ripening, regardless of hot water treatment or controlled atmosphere (Kim et al., 2007).

On the other hand, imazalil is a fungicide that inhibits the biosynthesis of ergosterol, an inhibitor of cytochrome P-450 enzyme that demethylates ergosterol (Pérez-Márquez et al., 2016). Imazalil acts directly on the fungus, affecting its cellular permeability and lipid biosynthesis reduces spore germination and the inflammation of the germ tube distortion and cytoplasm loss in germinated cells (Pérez-Márquez et al., 2016). In addition, the application of essential oil (a mix of eugenol, menthol, and carvacrol), decreases cellular respiration in the fruit and the production of ethylene (Guillén et al., 2012).

Although thermal-processing techniques inactivate microorganisms and spoil enzymes, inadequate handling of heat-processing methods may induce several chemical changes in the fruit and reduces not only the content or bioavailability of some phytochemical compounds but also the organoleptic properties (Patras et al., 2010).

For example, Beyers and Thomas (1979) showed that blanching at 80°C for 5 min of mango fruit produces loss of carotenoids. Kim et al. (2009) also evidenced that total soluble phenolics, gallic acid, and gallotannins of mango fruit diminished as a result of prolonged hot water treatment (46°C for 70 to 110 min).

On the contrary, Keitt mango fruit treated with hot water dipping at 50°C for 30 min had slightly lower content of TSS until 6 days of storage, but after 9 days of storage, the final value of TSS was higher than at the beginning of the experiment, and the acidity was not significantly affected by the heat treatment (Djioua et al., 2009). Hot water dipping at 50°C for 30 min and 46°C for 75 min maintained the contents of vitamin C until 3 days of storage, whereas at 46°C for 30 min and 50°C for 75 min, the content of vitamin C quickly decreased (Djioua et al., 2009). These results suggest that with a proper time–temperature combination, these losses can be diminished, and fresh-cut products can still contain important levels of vitamin C during storage (Djioua et al., 2009). Finally, the carotenoid content of Keitt mango treated with hot water (46°C/75 min, 50°C/30 min, and 50°C/75 min) increased because of disruption of cell membrane induced by heat treatments, and extraction enhanced and chemically enhanced extraction of carotenoids; moreover, during storage, total carotenoid contents remained stable compared with those in non-treated fruit, which could increase the antioxidant capacity (Djioua et al., 2009).

Currently, the methods employed to extend the shelf life of mangoes include physical and chemical treatments to reduce respiration and ethylene production, but the storage techniques are expensive and not fully satisfactory and may lead to the development of off-flavor if temperatures used are lower than the optimum.

Treatment with plant hormones, such as methyl jasmonate, and their synthetic derivatives are used to improve mango fruit quality, to enhance color uniformity, to increase the activity of phenylalanine ammonia lyase, and to increase the content of total phenolic compounds including anthocyanins (Lalel et al., 2003a; Lalel et al., 2003b; Lalel et al., 2003c). They also improve β-carotene, vitamin C, glucose, fructose, and sucrose contents and the ratio of soluble solids/titratable acidity, increase firmness, and reduce weight loss (Muengkaew et al., 2016). Other treatments such as 1-methylcyclopropene (1-MCP) are used commercially to retard ripening, while ethrel is used to accelerate. 1-MCP treatment decreases lipid peroxidation and increases activities and isozymes of catalase and superoxide dismutase in Dashehari mango, while ethrel treatment has opposite effects (Singh and Dwivedi, 2008). It was observed that the use of 1-MCP in Kensington Pride cultivar at higher concentrations (10 and 25 μl/L) affect the production of monoterpenes, esters, aldehydes, and aroma volatile compounds, whereas application of low concentrations of 1-MCP (1 μl/L) has less effect on the aroma profiles of this mango cultivar (Lalel et al., 2003a; Lalel et al., 2003b; Lalel et al., 2003c). In relation to chemical treatments to slow fruit softening, the addition of divalent calcium ions solutions promotes the formation of calcium bridges between the pectic polysaccharide chains (Tharanathan et al., 2006).

The non-thermal-processing technologies such as irradiation has been used as treatments for preservation, causing minimal modifications to the quality attributes of food, but might affect levels of nutrients and phytochemical compounds conditioned to the dose and radiation source used (i.e., gamma, X-ray, UV, and electron beam) (Bhat and Sridhar, 2008). Reyes and Cisneros-Zevallos (2007) evaluated the effect on the total phenolic, carotenoids, and vitamin C content of Tommy Atkins mango after electron-beam ionizing radiation in a dose range of 1–3.1 Gy and storage. They observed that flavonols after 18 days in storage (3.1 Gy) increased, total phenolics were not affected, but vitamin C decreased around 50–54% during storage (≥1.5 kGy), but no important alterations in carotenoid content were reported, indicating a retardation in ripening of treated mangoes (1–3.1 kGy) compared with non-irradiated fruits. However, at the lowest dose (≥1.5 kGy), flesh pitting was observed, indicating presence of death tissue due to the induced oxidative stress (Reyes and Cisneros-Zevallos, 2007). Cruz et al. (2012) compared the effect of irradiation at 0.4 and 1.0 kGy with the hot water dipping treatment (46°C for 90 min) on organic acid content, among other attributes of Tommy Atkins mangoes from Brazil for export stored for 14 days at 11°C and then 23°C until the end of the study. They reported that the higher levels of citric and succinic acids were present in the control group (untreated fruit) on the last day of the study. In addition, no significant differences in the total sugar content were observed between groups (control vs. treated); finally, gamma radiation does not seem to negatively affect the quality attributes of mangoes.

At present, molecular biology techniques are good options for the control of ripening, like the antisense RNA technology, which signifies a single type of DNA transcript of 19–23 nucleotides and is complementary to mRNA (Xu et al., 2018). This RNA technology has been used to regulate gene expressions during the replication, transcription, and translation. For example, antisense RNA of 1-aminocyclopropane-1-carboxylate oxidase inhibits the expression of rate-limiting enzymes involved in the biosynthesis of ethylene in tomatoes, delaying its maturation (Oeller et al., 1991). At present, ongoing studies have been applied to mangoes, among other fruits (Xu et al., 2018).

## Conclusions and Perspectives

Mango is a valuable fruit from a nutritional point of view, providing fiber, micronutrients as carbohydrates (10–32% in ripe pulp), proteins (0–5%), amino acids (alanine, arginine, glycine, serine, leucine, and isoleucine), lipids (0.75% to 1.7%), and organic acids (citric is the major organic acid, 0.13% to 0.71% FW).

Mango fruit also provides macronutrients such as vitamins (vitamin C, from 9.79 to 186 mg/100 g of mango pulp; vitamin A, from 1,000 to 6,000 IU; E and K vitamins are found in minor quantities; D vitamin has not been detected in any cultivars until now). Except for biotin, all the other B vitamins have been found in mango fruit. In addition, mango fruit is an important source of polyphenols (catechins, quercetin, kaempferol, rhamnetin, anthocyanins, tannic acid, and mangiferin; carotenoids, organic acids, and volatile compounds), useful for medicinal applications and also as indicators of fruit quality. All these concentrations depend on ripe state of the mango pulp and peel.

This review shows changes in nutritional and phytochemical components during postharvest handling practices, such as trimming, delatexing/desapping, sorting/grading, and disease control. Knowing and understanding the changes in the chemical composition in mango fruit during its development will allow producers to better characterize their cultivars and select those that have phytochemical characteristics that give added value to the fruit, for example, enhancing fruit color, delaying the maturation process, selecting fruit with a greater contribution of certain nutrients, increasing antioxidant components, and improving fruit characteristics for export purposes or greater use for agro-industry and processing.

## Author Contributions

MEM-C and EY collected the literature; wrote the sections related to nutritional composition, phenolic and pigments compounds, and the changes of nutritional and phytochemical components during the postharvest process of the fruit; made critical edits; and reviewed the whole manuscript before submission. RB collected the literature, wrote the section on carbohydrates, and prepared the figures presented. PL and JCGO collected the literature and wrote the sections related to volatile compounds and organic acids. BR collected the literature and contributed to the section on phenolic compounds. NL and JA collected the literature and wrote the sections related to changes of the nutritional and phytochemical compositions during growth and ripening.

## Funding

This work was funded by the Francisco José de Caldas Institute for the Development of Science and Technology (COLCIENCIAS), Ecosistema Científico Convocatoria 778 de 2017 (grant number FP44842-211-2018), Estrategia de Sostenibilidad de Grupos 2018-2020 de la Escuela de Nutrición y dietética from Universidad de Antioquia (Colombia), and the Consejo Nacional de Ciencias y Tecnología (CONACyT) de México.

## Conflict of Interest Statement

The authors declare that the research was conducted in the absence of any commercial or financial relationships that could be construed as a potential conflict of interest.
